# Some reflections on the use of inappropriate comparators in CEA

**DOI:** 10.1186/s12962-020-00226-8

**Published:** 2020-08-27

**Authors:** José Antonio Sacristán, José-María Abellán-Perpiñán, Tatiana Dilla, Javier Soto, Juan Oliva

**Affiliations:** 1 Department of Preventive Medicine and Public Health, School of Medicine, Universidad Autónoma de Madrid, Avenida Arzobispo Morcillo s/n. 28029, Madrid, Spain; 2 Medical Department, Lilly, Madrid, Spain; 3 Universidad de Murcia, Murcia, Spain; 4 Universidad Carlos III, Madrid, Spain; 5 Universidad de Castilla La Mancha, Toledo, Spain

**Keywords:** Incremental cost-effectiveness ratio, Efficiency, Economic evaluation, Threshold, Social perspective, Comparator, Health technology assessment

## Abstract

Although the choice of the comparator is one of the aspects with a highest effect on the results of cost-effectiveness analyses, it is one of the less debated issues in international methodological guidelines. The inclusion of an inappropriate comparator may introduce biases on the outcomes and the recommendations of an economic analysis. Although the rules for cost-effectiveness analyses of sets of mutually exclusive alternatives have been widely described in the literature, in practice, they are hardly ever applied. In addition, there are many cases where the efficiency of the standard of care has never been assessed; or where the standard of care has demonstrated to be cost-effective with respect to a non-efficient option. In all these cases the comparator may lie outside the efficiency frontier, so the result of the CEA may be biased. Through some hypothetical examples, the paper shows how the complementary use of an independent reference may help to identify potential inappropriate comparators and inefficient use of resources.

## Introduction

The aim of cost-effectiveness analysis (CEA) of health care programmes is to help policy makers to allocate scarce resources among available alternatives in order to maximize health outcomes [1]. Additional costs generated by one intervention over another are compared to the additional quality-adjusted life-years (QALYs) yielded, in the form of an incremental cost-effectiveness ratio (ICER). Decision rules have been developed to maximize the amount of QALYs provided by health care interventions restricted to a finite budget [2, 3]. According to the “fixed budget rule” [4] or “league table” approach [5], health care interventions are ranked in increasing order of ICER and then successively included in the health benefit basket or national health insurance scheme until the budget is exhausted. The ICER of the least cost-effective intervention that is adopted indicates the “critical ratio” [6] or cost-effectiveness threshold representing the opportunity cost of funding new programmes. On the contrary, according to the “fixed ratio rule” [4] or “threshold approach” [7], a new intervention is adopted if its ICER does not exceed a certain cost per QALY gained threshold of fixed price cut-off point. Both decision rules are coincidental if the budget implicitly determined by the “fixed ratio rule” is the same as the budget constraint assumed in the “fixed budget rule” [8].

In different countries, reimbursement and pricing decisions for new medicines are based on explicit or implicit cost per QALY thresholds [9–14]. Different league *tables* have been published attempting to rank-order an assortment of health interventions by cost-effectiveness [15–18]. Also, different methodological guidelines provide “reference cases” or “good practice codes” that CEA studies should follow to promote comparability among them [13, 19, 20]. Likewise, health technology assessment agencies have published reimbursement submission guidelines setting recommendations to conduct economic evaluations [21, 22].

Although CEA ‘s results may be affected by different assumptions, such as the rate at which future costs and benefits are discounted or the analysis’ perspective, the choice of the comparator is one of the main factors that influences the CEA’s results to a greater extent [23]. ICER is a relative concept in which the incremental costs and incremental effects of the analysis depend on the selected comparator (or the starting point of the analysis). The inclusion of an inappropriate comparator may introduce biases on the outcomes and the recommendations of an economic analysis. In this article, we describe the limitations of using inappropriate comparators, its impact on an inefficient use of resources and we propose a potential solution to identify the issue.

## Description of the problem: CEA results depend on the starting point of the comparison

ICER results may guide decision making between mutually exclusive alternatives (one patient can only receive one of the treatments for one indication; e.g. an antiulcer drug) or between independent treatment alternatives (e.g. breast cancer screening, oral anticoagulants, vaccination campaigns, etc.), each of which, in a turn, can encompass a set of several mutually exclusive alternatives. Most CEAs are conducted between mutually exclusive alternatives. Working on the efficiency frontier (the line on the cost-effectiveness plane connecting the non-dominated treatment alternatives) is the right way to calculate the cost-effectiveness of mutually exclusive interventions. Although the theoretical rules for cost-effectiveness analyses of mutually exclusive alternatives have been widely described in the literature, in practice, they are hardly ever applied: not all the mutually exclusive alternatives are systematically identified nor ranked according to ICER; strongly dominated and extended dominated alternatives are not always excluded; and there is not a formal process to identify and incorporate the most efficient alternatives into the health care system.

A review of 29 pharmacoeconomic guidelines [24] concluded that the most recommended comparator (in 86% of the guidelines) was “*the standard of care for local practices*” (assuming this is the alternative that would be replaced by the new intervention). However, very often, health care decision makers select a standard of care that is not an efficient alternative itself (e.g. a treatment for a severe disease, or for a rare disease, etc.). In addition, there are many occasions where the efficiency of the standard of care has never been assessed; or where the standard of care has demonstrated to be cost-effective versus a non-efficient option. In all these cases the result of the CEA could be biased as the new intervention could seem cost-effective versus another (in relation to a predefined threshold), when in fact it is an inefficient intervention.

The potential bias not only occurs in the case of mutually exclusive alternatives but also in the evaluation of independent treatments. Although independent interventions (vaccination, screening, etc.) are not mutually exclusive, they always compete for a limited health care budget. The relevant question here is: if the ICER of intervention A vs A´ is $20.000 per QALY and the ICER of intervention B vs B’ is $40.000 per QALY (both are efficient interventions considering a threshold of $50.000 per QALY), can we compare the ICERs of both interventions in the same league table if their starting point is different?

In summary, assuming than the standard of care (or the starting point) is always the right comparator for a CEA poses three important limitations. Firstly, the identification of the optimal intervention (i.e. the one deemed most cost-effective) may vary depending on the starting point for the analysis [25]. The addition (or the subtraction) of an alternative may lead to a change in the preference for the alternatives in the original set. This preference reversal challenges a very basic normative requirement of rationality known as invariance, extensionality or independence of irrelevant alternatives [26–29] according to which “supposedly irrelevant factors”, such as the content of the set of options among which the decision-maker has to choose, should not affect the preference order.

Secondly, it is frequently assumed that the standard of care is an efficient intervention, ignoring if the existing interventions against that condition are themselves worth doing [30]. This is equivalent to take for granted that the current mix of interventions are efficient when indeed probably “the starting point is the historical inheritance of a set of insured interventions whose evidential base was poor or left unexplored, many of which were selected for reasons other than a plausibly demonstrated highly effective impact on population health” [31].

Lastly, “the standard of care” (and hence the starting point of the comparisons) differs greatly from one therapeutic area to another, whilst ICERs are valued equal irrespective their origin. These differences are diverse and not always obey to efficiency reasons.

For example, in the area of oncology, many existing treatments are marginally better and much more expensive that the last treatment used as a comparator. In this case, it may be relatively easy for a new drug to demonstrate a favorable ICER compared to an inefficient standard of care [32]. On the other hand, in areas where only an old low-cost treatment (somewhat less effective than the new intervention) exists it may be difficult for a new intervention to demonstrate an acceptable ICER. In some way, the attractiveness of a therapeutic option is enhanced by the scope of the area to which it belongs to, what resembles a sort of contextual effect [33].

## Potential implications of using an appropriate comparator

The problem described in the previous section may have a significant impact on the efficient assignment of health care resources. In theory, resources in the health sector should be allocated across interventions and population groups in order to increase the population health. If, as in the case of mutually exclusive interventions, the standard of care is not an efficient intervention (or if it seems efficient compared to a non-cost-effective treatment); or, in the case of independent interventions, the starting point of the analyses generates non-comparable ICERs, the consequence would be an inefficient allocation of health resources.

It would be helpful to develop a tool to identify potential inappropriate comparators in CEA. The use of an independent reference (like the meter as the unit of length in the decimal system) is a possible solution. For example, the development of a “generalized CEA” was proposed by WHO [30] to assess the costs and benefits of each set of mutually exclusive and independent interventions with respect to the “do-nothing” option. In that way, the cost-effectiveness of all the interventions, including currently funded interventions, would be assessed applying the classical process of decision rules for CE analysis starting from the origin.

This paper does not propose a new methodology to conduct CEA, but a system to identify potential biased CEA due to the use of inappropriate comparators. Specifically, this work proposes the *complementary* use of an independent reference (an “independent” or “reference ICER”) to identify potential deviations of the “conventional” (context-dependent) ICER from the reference baseline. A high discrepancy (deviation) between both measures could indicate the existence of an inefficient use of resources. Although our approach is similar to the “generalized CEA” by the WHO, we propose that the costs and benefits of the interventions are not evaluated with respect to the counterfactual of the null set of interventions (i.e. doing nothing), but with regards to a selected baseline which could be similar to the ICER corresponding to some efficient public health interventions (e.g. $20,000/QALY or less). Next sections compare the results of the “conventional ICER” (calculated versus the standard of care) and those obtained using the “independent ICER” (calculated versus an independent comparator).

## Outline of the approaches to set up the comparator

Let ${p}_{i}$ stands for a typical programme to be evaluated from the set of available interventions $P=\left({p}_{1 },{p}_{2},\dots ,{p}_{n }\right).$ Programme *i* is characterized as a pair $\left({C}_{{p}_{i}},{QALY}_{{p}_{i}}\right)$ where ${C}_{{p}_{i}}$ and ${QALY}_{{p}_{i}}$ denote, respectively, the monetary cost and the number of QALYs attached to intervention ${p}_{i}$.

Let ${d}_{i}$ be the condition or disease-specific comparator (i.e. the current practice) with which programme ${p}_{i}$ is compared, in such a way that each intervention in set *P* has its related comparator, so $D=\left({d}_{1 },{d}_{2},\dots ,{d}_{n}\right)$. Disease-specific comparator *i* is characterized as a pair $\left({C}_{{d}_{i}},{QALY}_{{d}_{i}}\right)$.

Let *r* be a reference or independent comparator common to all the programmes belonging to set *P*. Reference or context-independent comparator *r* is described as the pair $\left({C}_{R},{QALY}_{R}\right)$.

The ${ICER}_{\left({p}_{i},{d}_{i}\right)}$ represents the additional monetary cost for each additional QALY obtained with an intervention ${p}_{i}$ over another programme ${d}_{i}$, calculated as follows:

$${ICER}_{\left({p}_{i},{d}_{j}\right)}=\frac{\left({C}_{{p}_{i}}-{C}_{{d}_{i}}\right)}{\left({QALY}_{{p}_{i}}-{QALY}_{{d}_{i}}\right)}$$

The ${ICER}_{\left({p}_{i},r\right)}$ of an intervention ${p}_{i}$ over the reference comparator *r* is computed as:

$${ICER}_{\left({p}_{i},r\right)}=\frac{\left({C}_{{p}_{i}}-{C}_{r}\right)}{\left({QALY}_{{p}_{i}}-{QALY}_{r}\right)}$$

Lastly, the indicator of the departure degree from the “incremental” rule (i.e. the adoption of the standard ICER, which is calculated with reference to the next best alternative) if the independent baseline *r* was used, ${I}_{\left({p}_{i},d,r\right)}$, is defined by:

$${I}_{\left({p}_{i},d,r\right)}=\left(\frac{{ICER}_{\left({p}_{i},r\right)}}{{ICER}_{\left({p}_{i},{d}_{j}\right)}}-1\right)\cdot 100$$

when ${I}_{\left({p}_{i},d,r\right)}$ = 0% then both types of evaluation—that based on a disease-specific comparator and that based on a context-independent comparator—agree. On the contrary if ${E}_{\left({p}_{i},d,r\right)}$ ≠ 0% a discrepancy emerges which should be considered by the decision-maker.

## Some hypothetical examples

Table 1 shows the costs and outcomes of various hypothetical programmes. Assume firstly that these programmes are not mutually exclusive, but independent ones, so there is a different disease-specific comparator for each of them. In this way, for example, intervention $${p}_{1}$$ could be a screening test, $${p}_{2 }$$ a pharmacological treatment, $${p}_{3}$$ a vaccination campaign, and so on. Next, also assume that their ICERs (expressed in terms of dollars per QALY gained) have been calculated by using disease-related comparators. Lastly, assume that a cost-effectiveness ratio of $50,000 per QALY is considered as a threshold for efficiency.

**Table 1:** Conventional incremental cost-effectiveness ratio (ICER) of five new programmes by using five disease-specific comparators

New healthcare intervention			Disease-specific comparator			
	Cost	QALY	Cost	QALY	ICER	Order
${p}_{1}$	30,000	0.8	28,000	0.4	5000	1
${p}_{2}$	30,000	0.8	28,000	0.7	20,000	2
${p}_{3}$	30,000	0.8	12,000	0.7	180,000	3
${p}_{4}$	60,000	0.8	58,000	0.4	5000	1
${p}_{5}$	90,000	0.8	88,000	0.7	20,000	2

The first three interventions have the same cost ($30,000) and generate the same health benefit (0.8 QALY). Option $${p}_{1}$$ has a very favorable ICER ($5000 per QALY gained) because its cost is marginally higher than that of the comparator ($28,000) and the benefit improves twofold (0.4 QALY). Intervention $${p}_{2}$$ is also efficient, although in this case, its cost ($28,000) and benefit (0.7 QALY) are just marginally better than the comparator. Intervention $${p}_{3}$$ is very inefficient ($180,000 per QALY gained), given that its cost is significantly higher than that of its comparator ($12,000) and its additional benefit is only slightly better (0.1 QALY). Intervention $${p}_{4}$$ is as efficient as intervention $${p}_{1}$$, even though its cost is double ($60,000) and it generates the same benefits (0.8 QALY). Finally, intervention $${p}_{5}$$ is the most expensive intervention ($90,000) in the table, but it is also an efficient choice (equivalent to $${p}_{2}$$), given that its additional cost and QALYs are marginally higher than the alternative option. According to a threshold of $50,000/QALY, a decision-maker would recommend the use of all interventions except intervention $${p}_{3}$$.

Table 1 shows that the efficiency of a given health intervention does not depend only on its own cost and effectiveness, but on cost and effectiveness of the alternative with which it is compared as well. These results cast several questions. For example, is it really more efficient intervention ${p}_{5}$ than intervention ${p}_{3}$, when the cost per QALY of the former is three times higher than that of the later? Or, are actually interventions ${p}_{1}$ and ${p}_{4}$, and interventions ${p}_{2}$ and ${p}_{5}$, equivalent in terms of efficiency?

The answer to above questions is that it depends. For example, a high cost intervention like ${p}_{5 }$ may seem very efficient because both its effectiveness and cost are just marginally higher than those of the comparator, which is indeed inefficient in comparison to the predefined threshold. Or because the comparator, though is not cost-effective, was reimbursed thanks to other factors distinct from ICER like the burden of disease or the rarity of the disease. Alternatively, an intervention such as ${p}_{3}$ could appear inefficient because the only available alternative (much cheaper and somewhat less effective) for that indication is an off-patent drug which was approved many years ago.

As noted in the Introduction, our point is that there are potential contextual effects that can bias the comparison of different ICERs. One source of such biases is, for example, the speed to which “the standard of care” changes due to the innovative dynamism existing in each therapeutic area. We think that the comparison of all the interventions to a common (non-null) reference comparator would allow to control for the existing dispersion throughout therapeutic areas. The result obtained from these comparisons would be a qualitative input that decision-makers could consider in order to prevent a mechanical application of the conventional ICER rule that ignores the possible sources of biases. The reference baseline could be a highly efficient health public intervention or, instead, some accepted efficiency bound.

Let us now show how an independent reference comparator would work with the same five hypothetical interventions depicted in Table 1. The ICERs of those interventions when compared with a standard comparator are shown in Table 2. In this case, a cost-effectiveness ratio of $20,000/QALY has been chosen, although to facilitate calculations, an equivalent cost of $5,000 per 0.25 QALY gained is included in the table. Interventions $${p}_{1}$$, $${p}_{2}$$, and $${p}_{3}$$ are equally efficient, while options $${p}_{4}$$ and $${p}_{5}$$ are inefficient. As shown in the first right column, the ranking of efficiency presented in Table 2 is different from that displayed in Table 1, when disease-related comparators were used.

**Table 2:** Independent incremental cost-effectiveness ratio (ICER) of five new programmes by using a standard comparator

New healthcare intervention			Standard comparator			
	Cost	QALY	Cost	QALY	ICER	Order
${p}_{1}$	30,000	0.8	5,000	0.25	45,455	1
${p}_{2}$	30,000	0.8	5,000	0.25	45,455	1
${p}_{3}$	30,000	0.8	5,000	0.25	45,455	1
${p}_{4}$	60,000	0.8	5,000	0.25	100,000	2
${p}_{5}$	90,000	0.8	5,000	0.25	154,545	3

The relative divergence between both types of ICERs is shown in Table 3. Visual analysis of Table 3 allows to compare the conventional and independent ICERs. In the case of programs 1 and 2, both ICERs are below the efficiency frontier, which would suggest that the disease specific comparator is adequate. On the contrary, the discrepancies between both ICERs in programs 3, 4 and 5, could be indicating a potential bias derived from the use of an inadequate disease specific comparator. In the case of intervention *p*_*3*_, the discrepancy could be indicating that we are facing an apparently inefficient program (which is efficient when using the independent comparator), and in the case of the ${p}_{4}$ and ${p}_{5}$ programs, we would be facing an apparently efficient programs (which is inefficient when using independent comparator).

**Table 3:** Indicator of the divergence degree (%) between the “reference” or “independent” ICER and the conventional ICER

New programmes	${ICER}_{\left({p}_{i},{d}_{i}\right)}$	${ICER}_{\left({p}_{i},r\right)}$	${I}_{\left({p}_{i},d,r\right) }(\%)$
${p}_{1}$	5000	45,455	809
${p}_{2}$	20,000	45,455	127
${p}_{3}$	180,000	45,455	−75
${p}_{4}$	5000	100,000	1900
${p}_{5}$	20,000	154,545	673

Table 3 also shows the percentage of deviation from the conventional ICER when the independent comparator ($5000, 0.25 QALY) is used. In this example, the sign of the deviation of an apparent efficient intervention such as $${p}_{4}$$ and $${p}_{5}$$ (1900 and 673%, respectively) differs from the sign of the deviation of an apparent inefficient programme such as $${p}_{3}$$(−75%). Likewise, deviations of interventions sharing the same conventional ICER, such as $${p}_{1}$$ and $${p}_{4}$$(5000$/QALY), and $${p}_{2}$$ and $${p}_{5}$$(20,000$/QALY), are now quite different (deviation of $${p}_{4}$$ is more than double that that of $${p}_{1}$$ and deviation of $${p}_{5}$$ is more than five times that that of programme $${p}_{2}$$).

## Conclusion

The key message of this paper is that the inclusion of an inappropriate comparator may introduce biases on the outcomes and the recommendations of an economic analysis. As Mason et al. [34] assert:

“Decision makers should satisfy themselves that current practice is itself worth having before using it as a comparison for a new treatment. If the comparison programme is inefficient the analysis will be misleading”.

As the above examples show, different starting points can lead to different results in CEA. This bias violates basic rationality criteria in a similar way that contextual effects do in experiments on individual choices [35]. Apart from this problem, there are also significant differences in the speed to which innovation spreads in diverse therapeutic areas which makes difficult comparisons among them.

This paper proposes the adoption of a common baseline to which new healthcare interventions are compared to identify potential biases in the results of CEA. This baseline could be a highly efficient public health intervention. This information would be an “additional factor” to take into account in reimbursement recommendations. Our proposal differs from generalized CEA [30] in that the set of interventions are not evaluated with respect to the counterfactual of the null set. We are aware that there are different constraints that limit the possibility of reallocating resources across different therapeutic areas, but the comparison of all the interventions to the same independent comparator may help to identify ineffiencies between therapeutic areas. The result obtained from these comparisons would be an input to consider in order to prevent the automatic application of the ICER rule.

It is important to remark that the main objective of our proposal is not to replace the ICER for the ACER (average cost-effectiveness ratio), but to prevent contextual biases derived from using disease-specific comparators. The use of a common unit of measure, established by consensus, could contribute to consider the opportunity cost of including a new intervention and to adopt divestment decisions. We do not claim to overrule the context of marginal decisions. Rather, we claim for a correct implementation of marginal analysis avoiding starting-point biases and taking account concerns on the “historical inheritance” of the set of insured interventions in the different therapeutic areas.

## Data Availability

Not applicable.
